# The Potassium Utilization Gene Network in *Brassica napus* and Functional Validation of *BnaZSHAK5.2* Gene in Response to Potassium Deficiency

**DOI:** 10.3390/ijms26020794

**Published:** 2025-01-18

**Authors:** Xingzhi Qian, Hanrong Liu, Jie Zhou, Wenyu Zhu, Liping Hu, Xiaoya Yang, Xiwen Yang, Huiyan Zhao, Huafang Wan, Nengwen Yin, Jiana Li, Cunmin Qu, Hai Du

**Affiliations:** 1 College of Agronomy and Biotechnology, Southwest University, Chongqing 400716, China; qxz799252@163.com (X.Q.); hanrong.liu@ubc.ca (H.L.); zj893422105@163.com (J.Z.); zhuwenyu_0120@163.com (W.Z.); lipinghu0630@163.com (L.H.); 13118406296@163.com (X.Y.); yxw20000910@163.com (X.Y.); zhaohuiyan@swu.edu.cn (H.Z.); wanhua05@163.com (H.W.); nwyin80@swu.edu.cn (N.Y.); ljn1950@swu.edu.cn (J.L.); drqucunmin@swu.edu.cn (C.Q.); 2 Academy of Agricultural Sciences, Southwest University, Chongqing 400716, China

**Keywords:** *Brassica napus* L., potassium utilization gene network, gene function, *BnaZSHAK5.2*

## Abstract

Potassium, an essential inorganic cation, is crucial for the growth of oil crops like *Brassica napus* L. Given the scarcity of potassium in soil, enhancing rapeseed’s potassium utilization efficiency is of significant importance. This study identified 376 potassium utilization genes in the genome of *B. napus* ZS11 through homologous retrieval, encompassing 7 functional and 12 regulatory gene families. These genes are unevenly distributed across 19 chromosomes, and the proteins encoded by these genes are mainly localized in the cell membrane, vacuoles, and nucleus. Microsynteny analysis highlighted the role of small-scale replication events and allopolyploidization in the expansion of potassium utilization genes, identifying 77 distinct types of *cis*-acting elements within their promoter regions. The regulatory mechanisms of potassium utilization genes were provided by analyses of transcription factors, miRNA, and protein interaction networks. Under low potassium stress, the potassium utilization genes, particularly those belonging to the KUP and CBL families, demonstrate pronounced co-expression. RNA-seq and RT-qPCR analysis identified the *BnaZSHAK5.2* gene, which is a high-affinity potassium ion transporter, playing a crucial role in the stress response to potassium deficiency in *B. napus*, as its expression is strongly induced by low potassium stress. A functional complementation study demonstrates that the *BnaZSHAK5.2* gene could rescue the primary root growth of the *Athak5* mutant under low potassium conditions, confirming its role in response to low potassium stress by sustaining root development.

## 1. Introduction

Potassium (K) is one of the macro elements necessary for plant growth and development, accounting for 2–10% of plant dry weight [1], and is widely involved in various physiological and biochemical regulatory processes of plants. Moreover, potassium ions in plant vacuoles are pivotal for the maintenance of intracellular ion homeostasis, where they interact with anions in the vacuolar and cytosolic compartments, serving as efficient regulators. Additionally, the free form of potassium ions in the cytoplasm is integral to numerous physiological and biochemical processes throughout the plant’s growth cycle, which are essential for sustaining cellular metabolism. Potassium ion channels play a critical role in preserving normal plant metabolism and responding to environmental stresses, including the modulation of membrane protein activity, osmotic balance, charge neutralization, sugar co-transport, and the activation of enzymes and functional proteins [2]. Additionally, K can also regulate plant growth and differentiation, such as root development [3] and pollen tube elongation [4]. However, most K in the soil is in crystalline form [5], with 90–98% of K not available and cannot be directly used by plants [6]. Therefore, potash is extensively applied to ensure maximum crop yields in practice. However, the leaching phenomenon of soil and low K+ utilization efficiency in crops leads to the waste of potash, which not only increases production costs but also causes environmental pollution [7]. To reduce production costs and develop sustainable agriculture, it is important to improve the K+ utilization efficiency of crops, and the elucidation of the molecular mechanism behind the K+ utilization process in crops is crucial for this goal.

Since the K+ channel genes *AtAKT1* and *AtKAT1* were first cloned in *Arabidopsis* [8,9], great progress has been made so far in the cloning and identification of K+ utilization network genes in plants. There are at least two K+ uptake mechanisms in plants, namely K+ channel-mediated low-affinity K+ uptake and K+ transporter-mediated high-affinity K+ uptake [10,11]. To date, more than 70 types of K+ channels and transporters have been identified in the model plant *Arabidopsis* participating in the K+ utilization process, and they are mainly divided into three K+ channel protein families, namely Shaker, TPK (two-pore K+), and TPC (two-pore channel) [12,13,14] and three K+ transporter protein families, namely HKT (high-affinity K+ transporter), KT/HAK/KUP, and CPA (cation proton antiporters superfamily), which together form the K+ functional gene families in plants. Among them, the AtKC1 and AtAKT1 proteins interact to form the inward-rectifying isomeric K+ channel [15,16], participating in absorption under a wide range of external K+ concentration (~0.01~10 mmol/L) [17,18]. AtTPK1 is located on vacuole membranes and is one of the most characteristic members of the TPK family, playing an important role in cellular K+ homeostasis, K+ release during stomatal closure, and the seed germination process [19,20]. *AtHAK5* still functions under external 0.01 mmol/L K+ concentration in Arabidopsis [21], and *OsHAK1* transcripts accumulate mainly in the root of K+ deficiency in *Oryza sative* [22]. In addition to functional genes, a variety of regulatory genes were also found in regulating the expression of K+ channels and K+ transporters in plants, such as MYB, ARF, and AP2/ERF [23,24]. *AtRAP2.11*, a member of the AP2/ERF family, positively regulates *AtHAK5* expression under K+ starvation, while ARF family member *AtARF2* negatively regulates *AtHAK5*. The above functional genes and regulatory genes together constitute the K+ utilization gene network, determining K+ utilization efficiency in plants.

*B. napus* (genome AACC, 2n = 38), an allotetraploid originated via hybridization between *Brassica rapa* (genome AA, 2n = 20) and *Brassica oleracea* (genome CC, 2n = 18) about 7500 years ago [25], is an important oil crop in China. *B. napus* requires large amounts of K+ for growth and development, approximately 290–370 kg per hectare [26,27], and K+ deficiency will severely limit the growth of leaves and siliques, resulting in lower yields. However, the average level of potash utilization efficiency in *B. napus* production is only 39.3% [28]. Therefore, it is meaningful to elucidate the molecular mechanism of K+ utilization in *B. napus*, which is essential for improving K+ utilization efficiency.

In this study, we have categorized genes involved in both direct potassium uptake and those influencing potassium-related processes, such as auxin distribution, under the umbrella term of “K+ utilization genes”. We systematically analyzed the K+ utilization gene network at the genome-wide level and gained a deeper understanding of the distribution, evolution, and expression of K+ utilization genes in *B. napus*. We first identified and constructed the K+ utilization gene network in *B. napus* and explored its evolution and regulatory mechanisms. Furthermore, we explored the expression patterns of the K+ utilization network genes under low K+ (LK) stress and normal spatiotemporal development and further constructed a co-expression gene network based on the LK stress expression profile and spatiotemporal development expression profile, respectively. Combined with differential expression analysis, the core genes of LK stress response in the K+ utilization network gene of *B. napus* were further explored. Additionally, through an integrated approach involving bioinformatics analysis and real-time quantitative PCR (RT-qPCR), we identified a candidate gene, *BnaZSHAK5.2*. We successfully cloned the coding sequence (CDS) of this gene, along with the upstream 1656 bp promoter region. To delve into the molecular functions of this gene, we employed a gene functional complementation assay for systematic preliminary validation.

## 2. Results

### 2.1. Identification and Construction of K+ Utilization Network Genes in B. napus

We identified 376 K+ utilization network genes from 19 gene families in the *B. napus* ZS11 ecotype genome, including 242 functional genes involved in the K+ utilization network and 134 regulatory genes involved in regulating the process of K+ utilization. Among the K+ utilization network genes, 47 of the 242 functional genes are homologs from Shaker (27 genes), TPK (two-pore K+, 16 genes), and TPC (two-pore channel, four genes) K+ channel gene families; 186 of the 242 functional genes are homologs from HKT (high-affinity K+ transporter, 8 genes), KUP (K+ uptake permeases, 54 genes), and CPA (cation proton antiporters superfamily, 124 genes) K+ transporter gene families; and the other 9 genes are the homologs from NRT, which is the member of the Nitrate Peptide Transporter (NPT) gene family, facilitating the coordination of N/K+ translocation from root to xylem. The 134 regulatory genes are divided into 14 regulatory gene families, namely R2R3-MYB (12 genes), CPK (calcium-dependent protein kinase, 20 genes), CIPK ((CBL)-interacting protein kinase, 22 genes), CBL (calcineurin B-like protein, 34 genes), AP2/ERF (APETALA2/ethylene responsive factor, 13 genes), bHLH (basic helix–loop–helix, 2 genes), LBD (LATERAL ORGAN BOUNDARIES DOMAIN, 4 genes), ARF (auxin response factor, 7 genes), PP2C (PROTEIN PHOSPHATASE 2C, 4 genes), C2H2 (2 genes), 14-3-3 (5 genes), SNARE (soluble N-ethylmaleimide-sensitive factor, 4 genes), and TFIIA2 (5 genes) families.

**Figure 1:**
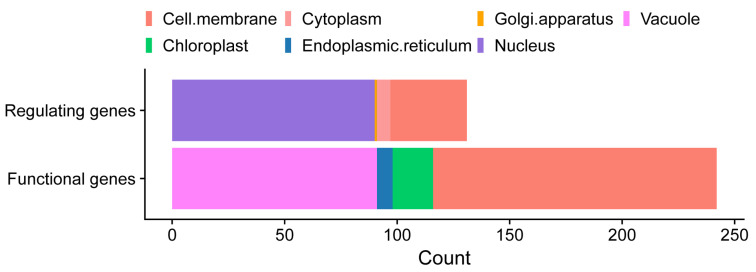
Subcellular localization of K+ utilization-related proteins in *B. napus*.

Subcellular localization analysis showed that functional proteins were mainly located in the cell membrane or vacuole while regulatory proteins were commonly located in the nucleus, with a small number of proteins located in the chloroplast, endoplasmic reticulum, cytoplasm, and Golgi apparatus (Figure 1). Additionally, the proteins located in the chloroplast are mainly K+ transporters *BnaTPKs* and *BnaKEAs*, and the proteins located in the endoplasmic reticulum and Golgi apparatus are mainly the K+ transporter *BnaKEAs* and regulatory protein *BnaSYP121*. In summary, K+ utilization network proteins were widely located in various organelles, while the differences in the subcellular localization of the functional proteins and regulatory proteins reflect their distinct roles and regulatory interactions within plant cells, contributing to overall potassium homeostasis and response to environmental cues. Proteins of the same gene family are often located in the same subcellular structure, indicating functional conservatism, while differences in subcellular localization between different subfamilies within a gene family indicate functional differentiation of genes.

### 2.2. Chromosome Localization, Duplications, and Evolution of K+ Utilization Network Genes in B. napus

**Figure 2:**
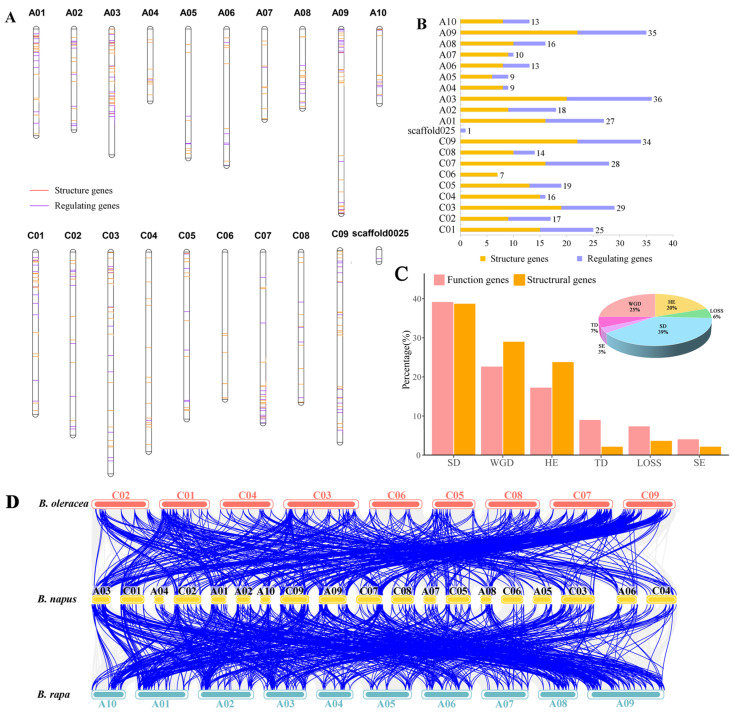
Analysis of gene evolution of K+ utilization-related genes in *B. napus*. (**A**): Chromosome mapping of K+ utilization genes. (**B**): Number of K+ utilization genes on each chromosome. (**C**): Gene duplication event, upper right corner shows duplication events of all K+ utilization genes, and bar chart shows duplication events of functional and regulatory genes of all K+ utilization genes. (**D**): Linear relationship analysis of *B. napus* K+ utilization pathway genes and homologous genes in *B. rapa* and *B. oleracea* genomes.

Chromosome localization analysis showed that, except one gene (*BnaMYB77.5*), the remaining 375 K+ utilization network genes were unevenly distributed on 19 chromosomes of *B. napus* (Figure 2A,B). Additionally, the distribution trends of genes in the An (186 genes) and Cn (189 genes) sub-genomes were similar, and the distribution of candidates along the chromosomes varies, with no clear trend towards accumulation at the physical location.

Collinearity analysis showed that 353 of the 375 genes had a syntenic relationship in at least one of the three genomes (*B. oleracea*, *B. rapa*, and *B. napus*) (Figure 2C,D). Among these 353 genes, 94 (~25%) were directly inherited from *B. rapa* (82; ~21.81%) and *B. oleracea* (12; ~3.19%) through the allopolyploid event. The remaining 259 genes were derived from small-scale duplication events, including segmental exchange (SE; 13 genes; ~3.46%), homologous exchange (HE; 74 genes; ~19.68%), segmental duplication (SD; 147 genes; ~39.10%), and tandem duplication (TD; 25 genes; ~6.65%). In summary, allopolyploid events and small-scale duplication events (especially SD events) contributed to the expansion of K+ utilization network genes in the *B. napus* genome. To further explore the genetic relationships within the *B. napus* genome, which is composed of AACC sub-genomes, we conducted intraspecific collinearity analysis, which provides insights into the evolutionary history and potential functional redundancy of genes within *B. napus* (Supplementary Figure S2).

Interestingly, 97.24% (318 genes) of the K+ utilization network genes had an An sub-genome background (*B. rapa* background). Among them, all of the SE events of K+ utilization network genes were replaced by the An to Cn sub-genome, and ~97.62% of the genes that were inherited from the An sub-genome were replaced by homologs from the Cn sub-genome via HE events, whereas ~92.63% of the genes in this network that were inherited from *B. rapa* were subsequently duplicated by SD events in *B. napus*, indicating that most of the K+ utilization genes were generally duplicated from the An sub-genome to the Cn sub-genome through small-scale duplication events. Additionally, genes derived from the *B. rapa* genome were more preserved on the An sub-genome in the allopolyploid events, while genes derived from the *B. oleracea* genome are less retained on the Cn sub-genome, indicating the increasing importance of the *B. rapa* genome background in *B. napus*.

Meanwhile, we identified 135 duplication genes in *B. napus* by collinearity analysis. Sequence identity analysis of these 135 duplication genes found that ~88.15% (119 genes) and ~87.41% (118 genes) of the duplication genes had a sequence identity ≥60% at the protein and CDS levels, showing sequence similarity and indicating less evolution in coding sequences. However, the identity is significantly reduced in the promoter regions, and the result showed that only ~56.3% (76 genes) of the duplication genes had sequence identity ≥60%. The neo-functionalization and sub-functionalization of duplication genes are influenced by a combination of changes in both coding sequences and promoter regions. While coding sequences are subject to strong evolutionary pressure and can significantly affect protein function, promoter regions also play a crucial role in regulating gene expression and can contribute to the divergence of duplicated genes.

Ka/Ks is used to determine whether there is selective pressure in the process of molecular evolution, and we calculated the Ka/Ks values of 90 sets of orthologous genes from 6 functional gene families and 14 regulatory gene families in *Arabidopsis*, *B. rapa*, *B. oleracea*, and *B. napus*, respectively (Supplementary Figure S1). We found that K+ utilization network genes are generally affected by purifying selection or neutral selection. However, these 90 sets of orthologous genes also have up to 612 positive selection sites (Ka/Ks > 1), and functional genes and regulatory genes contain 509 and 103 sites, respectively, indicating that functional genes have more non-synonymous mutation sites during evolution, and the function of most regulatory genes is relatively conserved.

### 2.3. Potential Transcriptional Regulation Profile of K+ Utilization Network in B. napus

**Figure 3:**
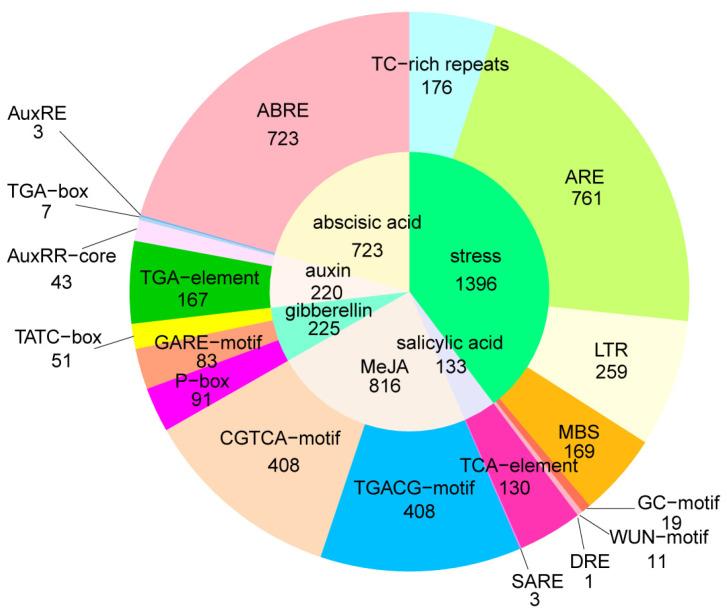
*Cis*-acting elements of K+ utilization genes in *B. napus*.

We identified 77 types of 27,076 *cis*-acting regulatory elements (CREs) in the promoter regions (−1500 bp) of the 376 K+ utilization network genes using PlantCARE online software (Figure 3). The majority of these CREs were core *cis*-elements (e.g., CAAT-box and TATA-box) and light-responsive *cis*-elements (e.g., G-box and GT1-motif). Meanwhile, many hormone-responsive and abiotic stress-responsive *cis*-elements were found in the promoter regions of the K+ utilization network genes. The abscisic acid (ABA)- (ABRE, 270 genes) and MeJA (methyl jasmonate)- (TGACG-motif and CGTCA-motif, 237 genes) responsive *cis*-elements were the significantly enriched CREs, indicating that the expression of K+ utilization network genes may be regulated by various hormones. Additionally, four main types of abiotic stress-responsive *cis*-elements, namely anaerobic stress (ARE; anaerobic induction element, 318 genes), low-temperature stress (LTR; low-temperature stress, 157 genes), stress (TC-rich repeats, 129 genes), and drought stress (MBSs; drought-induced MYB binding sites, 130 genes) were detected in many promoter regions of K+ utilization network genes.

We predicted 469 pairs of potential regulatory relationships between 74 types of miRNAs and 159 K+ utilization network genes via psRNATarget online software (Supplementary Figure S3). Among them, 109 pairs of regulatory relationships are derived from miRNA395, suggesting that this miRNA plays an important role in the transcriptional regulation of K+ utilization network genes in *B. napus*. We predicted that most miRNAs (e.g., miRNA6031 and miRNA159) have potential regulatory relationships on functional genes and regulatory genes in the K+ utilization network. Meanwhile, we found that miRNA6035 and miRNA395 only have targets in functional genes. These results revealed the potential regulatory effects of miRNAs on K+ utilization-related genes.

### 2.4. Spatiotemporal Expression Analysis of K+ Utilization Gene in ZS11

Using the public RNA-seq dataset, we constructed the spatiotemporal expression profile of the K+ utilization network genes in 90 *B. napus* samples across different developmental stages (Supplementary Figure S4). Among 376 candidate genes, 332 had detectable expression levels (TPM ≥ 1) in the tissue investigated, including 206 functional genes and 126 regulatory genes. Most of the functional genes were preferentially expressed in reproductive organs (e.g., siliques, flowers, and seeds) than vegetative organs (e.g., roots and stems), while most of the regulatory genes showed high expression levels no matter if they were in reproductive genes or regulatory genes. Notably, of the 44 genes that lacked detectable expression, 21 were mapped to the A sub-genome, with 23 mapped to the C sub-genome. Additionally, we found that ~80% (96 pairs) of the 120 duplicated pairs mentioned above shared conserved spatiotemporal expression patterns (|Pearson correlation coefficient| ≥ 0.6) in these two profiles, indicating their functional redundancy, while only ~20% (24 pairs) of them showed different spatiotemporal expression patterns (|Pearson correlation coefficient| < 0.6), suggesting these duplicated gene pairs had experienced expression divergence and the emergence of neo-functionalization and sub-functionalization of duplicated pairs during the evolution process in *B. napus*.

### 2.5. Co-Expression Analysis of K+ Utilization Network in B. napus Based on RNA-Seq Datasets

**Figure 4:**
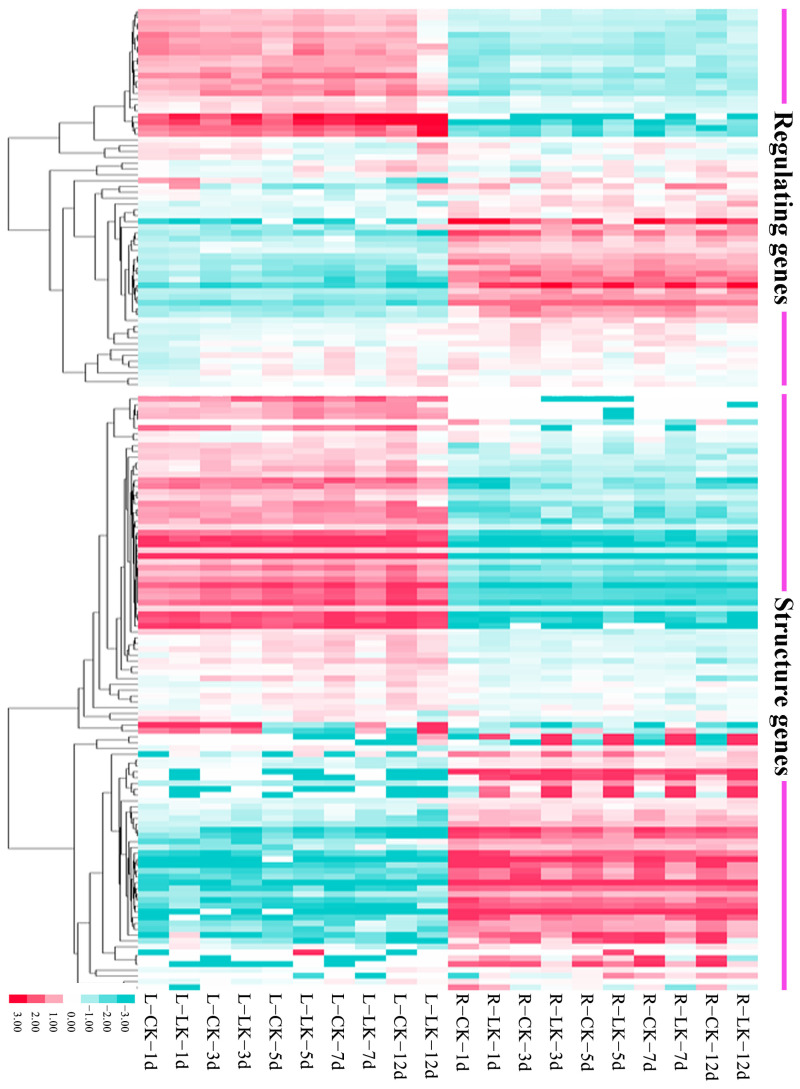
K+ deficiency stress expression profile of K+ utilization genes in *B. napus*. LK: K+ deficiency treatment. “1 d”, “3 d”, “5 d”, “7 d”, and “12 d” represent number of days after K+ deficiency treatment. L: leaf; R: root.

We used an RNA-seq dataset of the leaves and roots of the *B. napus* ZS11 ecotype at the seedling stage subjected to low potassium (LK) treatment to construct a potassium deficiency expression profile (Figure 4). The result shows 236 (~62.77%) of the 376 K+ utilization network genes showed detectable expression levels (FPKM ≥ 1) in roots and/or leaves, including 130 functional genes and 106 regulatory genes, while others showed no or weak (FPKM < 1) expression in the samples. Additionally, 129 of them were differentially expressed under LK treatment (FC ≥ 2, FDR < 0.01), including 58 functional genes and 49 regulatory genes. The expression of the two types of genes was divided into two opposite patterns, which were either preferentially and evenly expressed in roots or leaves, suggesting their different functions in the K+ utilization process. Among the 129 DEGs, 107 genes were upregulated, of which about 1/3 of the genes were members of the HKT, KUP, and CHX gene families, while only 22 genes were down-regulated, indicating they had different response trends during LK stress. Meanwhile, some genes were continuously expressed in roots or leaves regardless of whether they were under LK stress, such as BnaCDPK5.1, BnaGRF6.3, and BnaAKT1.3, suggesting their potential functional characteristics throughout the growth period of B. napus. In addition, we also found 13 genes (e.g., BnaHAK1 and BnaHAK5) that only had detectable expression levels under LK stress treatments, reflecting their irreplaceability in the case of extremely low concentrations of K+ in soil.

**Figure 5:**
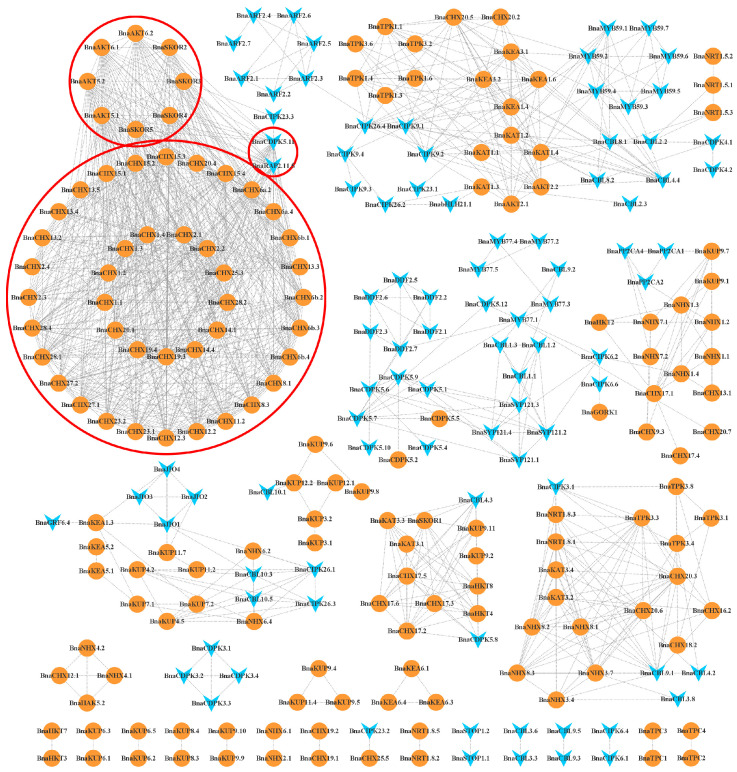
Co-expression network of K+ utilization genes in *B. napus* across different development stages. Orange circles represent transcription factors, and blue “V” shape graphics represent structural genes.

We conducted a co-expression analysis of K+ utilization network genes (Figure 5) based on the above two expression profiles (Supplementary Figure S4 and Figure 4). Co-expression relationship pairs with PCC (|Pearson correlation coefficient|) value ≥ 0.8 and *p*-value < 0.01 were obtained. In the spatiotemporal expression profile, we identified 1148 co-expression relationship pairs of 257 K+ utilization network genes, and its co-expression network has a distinct core structure. Its core structure consists of 49 functional genes and 2 regulatory genes, namely 41 CHX family members, 8 Shaker family members, 1 CDPK family member, and 1 ERF/AP2 family member, forming a complex gene network. Most of the genes in the core structure are mainly expressed in leaves and flowers during all developmental stages, with only a few genes expressed in roots. This indicates that K+ utilization network genes may play roles during the entire period of *B. napus* by coordinating with each other in temporal and spatial patterns.

In the LK stress expression profile, up to 6406 co-expression relationship pairs were identified in 215 K+ utilization network genes. Their core structures are mainly composed of 58 functional genes and 35 regulatory genes. Among them, the most numerous genes in functional genes and regulatory genes are members of the KUP and CBL families, respectively. These 93 genes can be divided into 2 categories: leaf expression only and root expression only, indicating that leaf cells regulate K+ transportation under LK stress and root cells absorb K+ in response to LK stress, respectively. However, there was no significant difference in the number of K+ utilization network genes expressed in leaves and roots, indicating that both leaves and roots were important organs responding to LK stress. In accordance with the results, members of the KUP family were found to be the dominant components of the core network of co-expression under conditions of K+ deficiency stress. In light of the findings of numerous functional studies on KUP family members, it was postulated that KUP proteins may play a pivotal role in K+ uptake and translocation, as well as in the response to LK stress in *B. napus*. Furthermore, spatiotemporal expression analysis revealed that genes with higher expression levels in roots also maintained high expression under LK stress, and some genes with initially lower expression levels showed an increase in expression under these conditions, further confirming the significant role of the KUP family in K+ absorption and response to LK stress.

### 2.6. Differential Expression Analysis of BnaZSKUPs Under K+ Deficiency Stress

**Figure 6:**
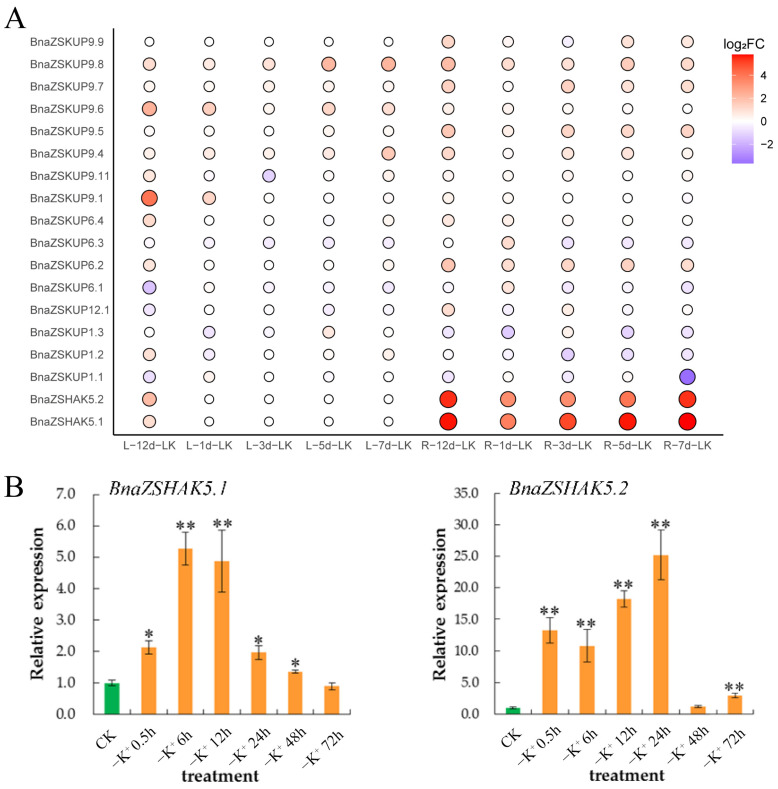
Gene expression pattern under potassium deficiency. (**A**): Differential expression of *BnaZSKUPs* under K+ deficiency stress. (**B**): Expression changes of *BnaZSHAKs* in potassium deficiency. * indicates that the *p* value is less than 0.5 and ** is less than 0.01.

According to the analysis of expression profiles of K+ utilization genes, the expression levels of five *BnaZSKUP*s increased and one *BnaZSKUP* decreased under K+ deficiency stress. Among them, *BnaZSHAK5.1* and *BnaZSHAK5.2* exhibited a significant upregulation trend from the first day of K+ deficiency treatment, reaching the highest differential expression levels in roots on days 7 and 12, respectively, after K+ deficiency stress (log2FC = 5.79 and 5.45, respectively) (Figure 6A). This suggests that these two genes may play an important role in potassium ion uptake under K+ deficiency conditions.

Subsequently, we employed quantitative real-time polymerase chain reaction (qRT-PCR) to further investigate the response mechanisms of these two genes under K+-deficient conditions in *B. napus* (Figure 6B). Our analysis indicated that both genes are highly sensitive to extracellular K+ ion concentrations, with their expression levels consistently increasing under K+ deficiency. This suggests that they are crucial for K+ absorption under such conditions. Moreover, the upregulation of *BnZSHAK5.2* was significantly more pronounced than that of *BnZSHAK5.1*, implying that *BnZSHAK5.2* may play a dominant role in response to K+ deficiency. Our findings suggest potential candidates for genes that may play a crucial role in the regulation of K+ transport under different physiological conditions. Future research could focus on the functional validation of these genes and their role in adapting to varying K+ availability, which could have significant implications for crop improvement and understanding nutrient use efficiency.

### 2.7. Functional Complementation of BnHAK5.2 Gene Restored Resistance of Arabidopsis Mutants to LK Stress

**Figure 7:**
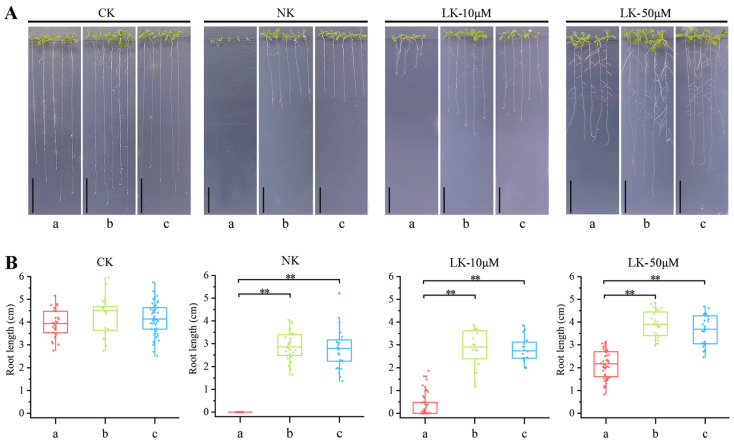
Phenotype analysis of root hair of transgenic *Arabidopsis*. (**A**): Phenotype of *Arabidopsis* root length. (**B**): Statistics of *Arabidopsis* root length. a: *athak5* mutant; b: WT; c: *BnaZSHAK5.2p::BnaZSHAK5.2*; LK: K+ deficiency; 10 μm and 50 μm represent concentration of K+ in medium; CK: full nutrition. ** indicates that the p value is less than 0.01.

In *Arabidopsis*, the *HAK5* gene was extensively studied, with its molecular functions and phenotypic characteristics elucidated in numerous research works [29,30]. In LK stress, the root length of *athak5* mutant plants was significantly shorter than that of wild type. The root hair length and density of overexpressed *AtHAK5* plants increased when they grew in a LK medium compared with the wild type. In order to investigate the function of the *BnaZSHAK5.2* gene, we generated a *BnaZSHAK5.2* functional complementary transgenic material of *athak5* (*BnaZSHAK5.2p::BnaZSHAK5.2 athak5*) and cultivated it alongside wild type (WT) *Arabidopsis* and the *athak5* mutant on Hoagland media varying in K+ concentrations (Figure 7). The *athak5* mutant exhibited heightened sensitivity to K+, with a pronounced reduction in root length under K+-deficient conditions. In stark contrast, the complementation line and the WT displayed no significant variation in root length across the spectrum of K+ concentrations tested. Despite the fact that the growth is inhibited, the vital functions of the root system were preserved, underscoring the critical function of *BnaZSHAK5.2* in sustaining root development under K+ stress and confirming that *BnaZSHAK5.2* has the same function as *AtHAK5*.

## 3. Discussion

### 3.1. Amplification and Evolution Mechanisms of K+ Utilization Genes in ZS11

Discussing the amplification and evolutionary mechanisms of K+ utilization genes in *B. napus*, we observed that these genes are primarily amplified through small-scale duplication events rather than whole-genome duplication (WGD) events, likely due to functional selection pressures from the environment [31,32,33]. Considering that *B. napus* is an allotetraploid resulting from the hybridization of *B. oleracea* (A genome) and *B. rapa* (C genome), both of which have undergone whole-genome duplication (WGD) events, it is expected that the gene content of *B. napus* would be approximately six times that of *A. thaliana*. Therefore, if Arabidopsis has 102 potassium utilization genes, *B. napus* would theoretically have 612 genes, but we identified only 376, indicating significant gene loss following the WGT event, with retention after the WGD event. Sequence identity analysis of 135 duplicated gene pairs revealed high conservation in coding sequences, but diversity in promoter sequences, suggesting functional diversification within the potassium utilization pathway of *B. napus* [34]. This diversity may stem from regulatory variations in promoter regions, such as differential transcription factor binding or chromatin structure alterations, potentially driving functional diversification. Retained duplicate genes often exhibit functional redundancy or undergo neo-functionalization and sub-functionalization, which could endow the organism with novel adaptive capabilities, while redundancy may act as a buffer against genetic perturbations, ensuring physiological stability.

### 3.2. Expression Patterns of K+ Utilization Genes in ZS11

In *B. napus*, the expression patterns of K+ utilization genes are intricately linked to their physiological roles [35]. Under K+ deficiency, approximately 62.77% of these genes are expressed in the roots or leaves of rapeseed, with 107 genes significantly upregulated, including 58 functional and 49 regulatory genes, highlighting their crucial role in potassium homeostasis. Notably, 44 of these functional genes encode K+ transport proteins involved in K+ transport under LK conditions. The upregulation of the remaining 14 K+ channel genes may be associated with increased synthesis of kinases such as CBL, CIPK, and CDPK, along with related transcription factors. *BnaHKT1* and *BnaHAK5.2* exhibit minimal expression under normal conditions but show high-level expression under K+ deficiency, indicating their high-affinity for K+. The HKT and KUP families predominantly mediate high-affinity K+ uptake in roots, with AtHAK5 being unique in its continuous function under low external K+ concentrations, as well as its homolog *OsHAK1* in rice [21,22]. Additionally, transcription factors *BnaRAP2.11.3*, *BnaRAP2.11.6*, *BnaDDF2.2*, and *BnaDDF2.6* enhance the transcription of *AtHAK5*, increasing K+ uptake under stress [23]. Our study identified 13 rapeseed K+ utilization genes that are exclusively expressed under K+-deficient conditions, suggesting specialized roles in K+ homeostasis. Conversely, a subset of genes maintains high expression in both roots and leaves, demonstrating low sensitivity to extracellular potassium concentration fluctuations, indicating their low-affinity nature. Approximately one-third of the genes, mainly from the Shaker, KUP, CBL, and CIPK families, are highly expressed in leaves but not in roots under abiotic stress conditions. These diverse expression profiles contribute to a sophisticated regulatory mechanism that enhances the efficiency of K+ utilization in *B. napus*.

### 3.3. The Critical Function of BnaZSHAK5.2 in K+ Stress Response

In *B*. *napus*, the critical role of the *BnaZSHAK5.2* gene in K+ stress response was revealed. Differential expression analysis of *BnaZSKUPs* under K+ deficiency has unveiled a complex regulatory network, with *BnaZSHAK5.1* and *BnaZSHAK5.2* emerging as key players in K+ uptake. The significant upregulation of *BnaZSHAK5.2*, in particular, suggests a central role in the plant’s response to K+ stress. qRT-PCR analysis further corroborates their sensitivity to extracellular K+ levels, indicating direct involvement in K+ absorption and homeostasis. Functional complementation of the *BnaZSHAK5.2* gene in Arabidopsis mutants provides a robust model to study its role in K+ stress tolerance. The stable root length of the complementation line across varying K+ concentrations underscores the gene’s critical function, contrasting sharply with the *athak5* mutant, which shows heightened sensitivity and reduced root length, underscoring the gene’s indispensable role in K+ acquisition. Our findings have significant implications for understanding the molecular mechanisms underlying potassium deficiency responses in plants, and the identification of *BnaZSHAK5.2* as a major player in K+ uptake and stress tolerance presents an opportunity for genetic manipulation to enhance crop resilience against K+ deficiency, a prevalent issue in agricultural production.

### 3.4. The Integral Role of Potassium in Root Elongation

The elongation of roots in *Arabidopsis thaliana* is a multifaceted process that involves the intricate interplay of several key cell types, particularly within the pericycle and vasculature, both of which are significantly influenced by potassium (K+). Our study, while centered on the role of root hairs in potassium uptake, also recognizes the pivotal significance of the pericycle and vasculature in the overall growth and development of the root system.

Potassium plays a crucial role in the pericycle, where it influences cell division and differentiation, leading to the formation of lateral roots and adventitious roots. Pericycle cells, under the influence of auxin, undergo division and differentiation to initiate the formation of root primordia, a process essential for the expansion of the root system and the plant’s ability to explore the soil for nutrients, including potassium. The vasculature, comprising the primary xylem and phloem, is responsible for the transport of water, minerals, and organic compounds. Potassium availability significantly impacts the maturation and function of the primary xylem, which matures in an exarch pattern, facilitating the upward movement of water and inorganic salts critical for cell expansion and root growth. Concurrently, the primary phloem develops to transport assimilates, supporting metabolic activities necessary for root elongation. The maturation and differentiation of cells within the vasculature, as well as the pericycle, are tightly regulated processes that ensure the root’s structural integrity and functional efficiency. The appearance of vascular tissues in the elongation zone, including the differentiation of sieve tubes and xylem vessels, indicates the root’s increasing capacity to support the plant’s nutritional and water requirements.

## 4. Materials and Methods

### 4.1. Identification and Subcellular Localization of K+ Utilization Genes in ZS11

To identify the potassium utilization gene families in *B. napus*, we conducted a comprehensive literature review of the PubMed database, focusing on genes associated with potassium utilization in *Arabidopsis* and *Oryza sativa* (https://pubmed.ncbi.nlm.nih.gov (accessed on 6 April 2024)) [15,16,19,20,21,22,23,36]. Subsequently, CDS sequences and protein sequences of these genes were obtained from the Phytozome website [37] (https://phytozome-next.jgi.doe.gov (accessed on 6 April 2024)). We then retrieved the protein sequences of these genes from the Phytozome database. Utilizing BLASTP, we performed a homology search against the genome database of the rapeseed variety “ZS11”, with an E-value threshold set to 1 using BlastP (http://cbi.hzau.edu.cn/cgi-bin/bnapus/blast (accessed on 6 April 2024)) [38,39]. The identified homologous genes were subjected to multiple sequence alignments using the MAFFT (https://mafft.cbrc.jp/alignment/server (accessed on 6 April 2024)) online tool, facilitating their classification into distinct gene families; sequences with large gaps and redundancies were excluded [40]. Through sequence similarity (>90%) and phylogenetic analysis, all homologous genes in the rapeseed potassium utilization pathway were ultimately determined.

To further ensure the accuracy of the data, in-depth analysis of the subcellular localization of rapeseed potassium utilization proteins was predicted using the Plant-mPLoc (http://www.csbio.sjtu.edu.cn/bioinf/plant-multi (accessed on 6 April 2024)) and WoLF PSORT (https://wolfpsort.hgc.jp (accessed on 6 April 2024)) online tools [41,42].

### 4.2. Chromosomal Localization and Collinearity Analysis of K+ Utilization Genes in ZS11

This study employed the GBROWSE tool from the BnPIR website (http://cbi.hzau.edu.cn/cgi-bin/bnapus/gb2/gbrowse/ZS11v0 (accessed on 6 April 2024)), to conduct a comprehensive analysis of the chromosomal localization of rapeseed K+ utilization genes [43]. Using MapChart2.3.2 software [44], we generated chromosomal localization maps to visually represent the distribution of these genes across the genome.

The One Step MCScanX plugin within the TBtool v2.056 software was used to perform an in-depth analysis and visualization of the collinearity between K+ utilization genes in ZS11 and those in the genomes of *B. rape*, *B. oleracea*, and *B. napus* itself [45]. By assessing the strength of collinearity relationships, we inferred the duplication events of these genes. Additionally, we created pie charts for a visual representation of the distribution of gene duplication events.

To obtain the protein sequences, CDS sequences, and promoter sequences of rapeseed K+ utilization genes, the BnPIR website was used once more. Subsequently, MatGAT2.01 software was applied to analyze the similarity and identity between duplicated gene pairs across protein, CDS, and promoter sequences, thereby evaluating the conservation of gene duplication [46].

### 4.3. Selection Pressure Analysis of K+ Utilization Genes in ZS11

In this study, we curated a set of 90 K+ utilization genes from *Arabidopsis*, excluding those without homologous genes. We employed MEGA5.1 software to align each *Arabidopsis* gene with its orthologs in *B. rape*, *B. oleracea*, and *B. napus* [47]. The alignment process involved the removal of stop codons and the deletion of unaligned sequence segments, followed by manual refinement to ensure the accuracy of the alignments. This resulted in 90 sets of orthologous gene alignments, which were saved in fasta format for further analysis. Subsequently, these alignment results were uploaded to the Datamonkey Adaptive Evolution Server (http://www.datamonkey.org (accessed on 6 May 2024)), where the FUBAR tool was used to calculate the ratio of non-synonymous to synonymous substitution rates (dN/dS values) for each set of orthologous genes [48,49]. This ratio is a critical indicator of selective pressure on genes. Finally, we used OriginLab Origin 2021 software to organize and visualize the computational results, providing a clear representation of the distribution and trends of selective pressures on the genes.

### 4.4. Functional Prediction Analysis of K+ Utilization Genes in ZS11

To elucidate the regulatory mechanisms of K+ utilization genes in ZS11, we utilized the PlantTFDB (https://planttfdb.gao-lab.org (accessed on 6 May 2024)) and PlantCARE (http://bioinformatics.psb.ugent.be/webtools/plantcare/html (accessed on 6 May 2024)) online platforms to conduct a comprehensive analysis of the promoter sequences (−1500 bp) of rapeseed K+ utilization genes [50,51]. This analysis aimed to predict potential transcription factor binding sites (*p*-value ≤ 1 × 10^−6^) and *cis*-acting elements. Furthermore, we submitted the CDS sequences of these genes to the psRNATarget website (https://www.zhaolab.org/psRNATarget (accessed on 6 May 2024)) to forecast possible miRNA regulatory relationships [52].

To visually represent these intricate regulatory networks, we employed Cytoscape 3.8.2 software for the visualization of the predicted outcomes [53]. Additionally, to investigate the interactions among rapeseed K+ utilization proteins, we uploaded the protein sequences to the STRING database (https://cn.string-db.org (accessed on 6 May 2024)) and constructed and edited the interaction network through this platform [54].

### 4.5. Expression Pattern Analysis of K+ Utilization Genes in ZS11

The spatiotemporal expression profiling of rapeseed K+ utilization genes was sourced from the BnTIR database (http://yanglab.hzau.edu.cn/BnTIR (accessed on 6 May 2024)) encompassing samples from eight distinct tissues/organs (cotyledons, roots, stems, leaves, buds, flowers, siliques, silique walls, and seeds) across three developmental stages (seedling, flowering, and podding) of the ZS11 cultivar, totaling ninety samples [55]. The transcriptomic data under K+ deficiency stress were generated by our research group and are not yet published; these data include root and leaf tissues at 5 time points (1, 3, 5, 7, and 12 days).

Leveraging the transcriptomic data obtained from the K+ deficiency stress in rapeseed, we focused on K+ utilization genes exhibiting maximum fragments per kilobase of transcript per million mapped read (FPKM) values greater than or equal to 1 across all samples for subsequent differential expression analysis. We employed the DESeq2 package to conduct this analysis, applying a threshold of log2 (fold change) ≥ 1 and *p*-value < 0.05 to identify significantly differentially expressed genes. Data processing and subsequent analysis were performed using Microsoft Excel 2021.

We employed the SYBR-Green PrimeScript RT-PCR kit (Beijing Baorui Medical Biotechnology Co., Ltd., Beijing, China) and the CFX Connect real-time system (Bio-Rad Laboratories, Chongqing, China) to conduct quantitative real-time PCR (qRT-PCR) analysis on the cDNA products. Each reaction was technically replicated three times, with *Arabidopsis thaliana Actin7* (GenBank accession number: XP_013678548.1) and *UBI* (*BnaC08g00780D*) serving as endogenous reference genes. The specific primers for the candidate genes are detailed in Table 1, using 2^−ΔΔCt^ methods to analyze gene expression.

### 4.6. Phenotypic Analysis of Transgenic Arabidopsis

In this study, we utilized the *p*EASY-*BnaZSHAK5.2* vector as a template and designed specific primers (F: 5′-ACAAAAATACAAAAAAGGGATCCGATGGATGGCGAGGAACATCA-3′; R: 5′-CACCATGTGTACTGTGTCGACCAGCTCATAAGTCATGCCAACCT-3′) to amplify the *BnaZSHAK5.2* gene fragment via PCR. Subsequently, the amplified fragment was digested with BamHI (N-terminal) and SalI (C-terminal) restriction enzymes, and the full-length CDS of *BnaZSHAK5.2* was cloned into the *p*BnaZSHAK5.2 expression vector using homologous recombination, resulting in the construction of the *p*BnaZSHAK5.2::*BnaZSHAK5.2* functional complementation vector. Then we conducted a phenotypic analysis on the *Arabidopsis* lines obtained, including the wild type (WT), *athak5* mutant, and the functionally complementary transgenic line (*pBnaZSHAK5.2::BnaZSHAK5.2 athak5*). The seeds were surface sterilized with 75% ethanol for 15 min, followed by thorough washing with sterile deionized water (ddH_2_O) 3–5 times. They were then sown on full nutrient Hoagland medium, potassium-deficient Hoagland medium, and Hoagland media supplemented with 10 μM and 50 μM K+, respectively. To create a potassium-deficient medium, we removed potassium nitrate (KNO_3_) from the standard medium. To maintain osmotic balance and ensure that the concentration of all other ions remained constant, we replaced KNO_3_ with an equimolar amount of calcium nitrate (Ca(NO_3_)_2_). This substitution allowed us to specifically study the effects of potassium deficiency on plant growth without confounding factors from changes in other nutrient levels. After 3 days of vernalization at 4 °C, the seedlings were cultivated in a controlled environment chamber; the specific conditions were set at an illumination intensity of 20,000 Lux, a relative humidity of 70%, and a 16 h photoperiod at 23 °C, followed by an 8 h dark period at 20 °C. This cultivation regimen is designed to mimic the natural light/dark cycle, allowing for the assessment of the growth performance of transgenic *Arabidopsis* under varying K+ supply conditions. The primary root length of each line was observed and measured.

## Figures and Tables

**Table 1:** Primer sequences of qPCR.

Genes	Sequence
*Actin7-F*	5′-AACCTTCTCTCAAGTCTCTGTG-3′
*Actin7-R*	5′-CCAGAATCATCACAAAGCATCC-3′
*ZSUBI-F*	5′-GCCATGGCTATTTTCCTTACAG-3′
*ZSUBI-R*	5′-GCCTCGTAGTCCAGATACTTTT-3′
*ZSHAK5.1-F*	5′-GAAACCGCTATACAAATGGCTACA-3′
*ZSHAK5.1-R*	5′-TGGTGTCACACGGCTCACG-3′
*ZSHAK5.2-F*	5′-GAGTTTGGCATTCCAGAGCCTA-3′
*ZSHAK5.2-R*	5′-TTGAGAAGTGCGACAAGGGTG-3′

## Data Availability

This study did not report any data.

## Supplementary Materials

The supporting information can be downloaded at: https://www.mdpi.com/article/10.3390/ijms26020794/s1.
