# Supplementary material for: The Potassium Utilization Gene Network in Brassica napus and Functional Validation of BnaZSHAK5.2 Gene in Response to Potassium Deficiency
Source: Int J Mol Sci. 2025 Jan 18;26(2):794. doi: 10.3390/ijms26020794 (PMC11765689; doi:10.3390/ijms26020794)
Supplement: Supplementary file 1 [file ijms-26-00794-s001.zip › Supplementary Figure S1.pdf]

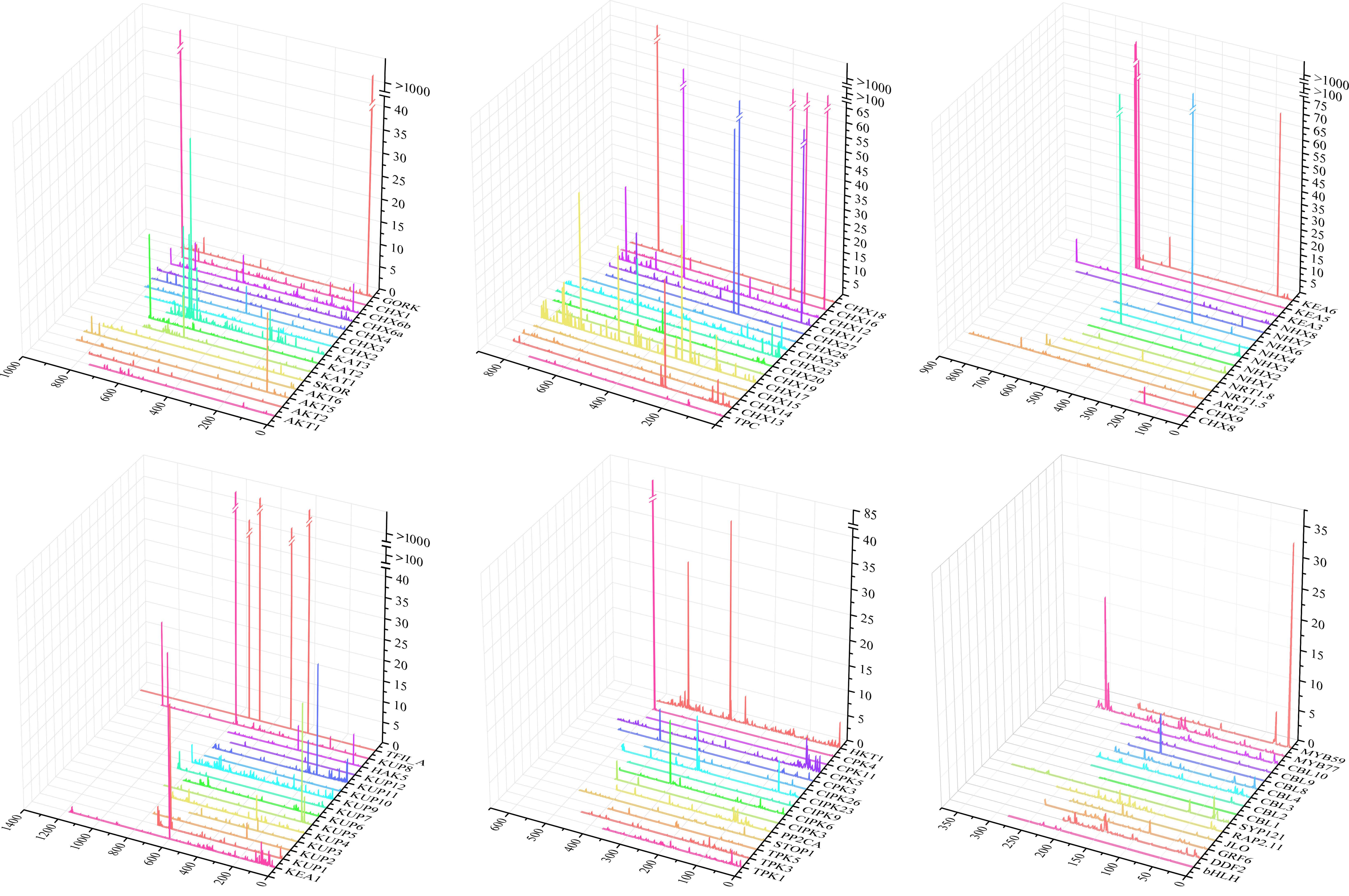

dN/dS analysis of K utilization genes in *B. napus*. The X-axis represents gene length; The Y-axis represents different types of orthologous genes from *Arabidopsis*, *B.rapa*, *B. oleracea* and *B. napus* (ZS11); The Z-axis represents dN/dS
