# Supplementary material for: The Potassium Utilization Gene Network in Brassica napus and Functional Validation of BnaZSHAK5.2 Gene in Response to Potassium Deficiency
Source: Int J Mol Sci. 2025 Jan 18;26(2):794. doi: 10.3390/ijms26020794 (PMC11765689; doi:10.3390/ijms26020794)
Supplement: Supplementary file 1 [file ijms-26-00794-s001.zip › Supplementary Figure S3.pdf]

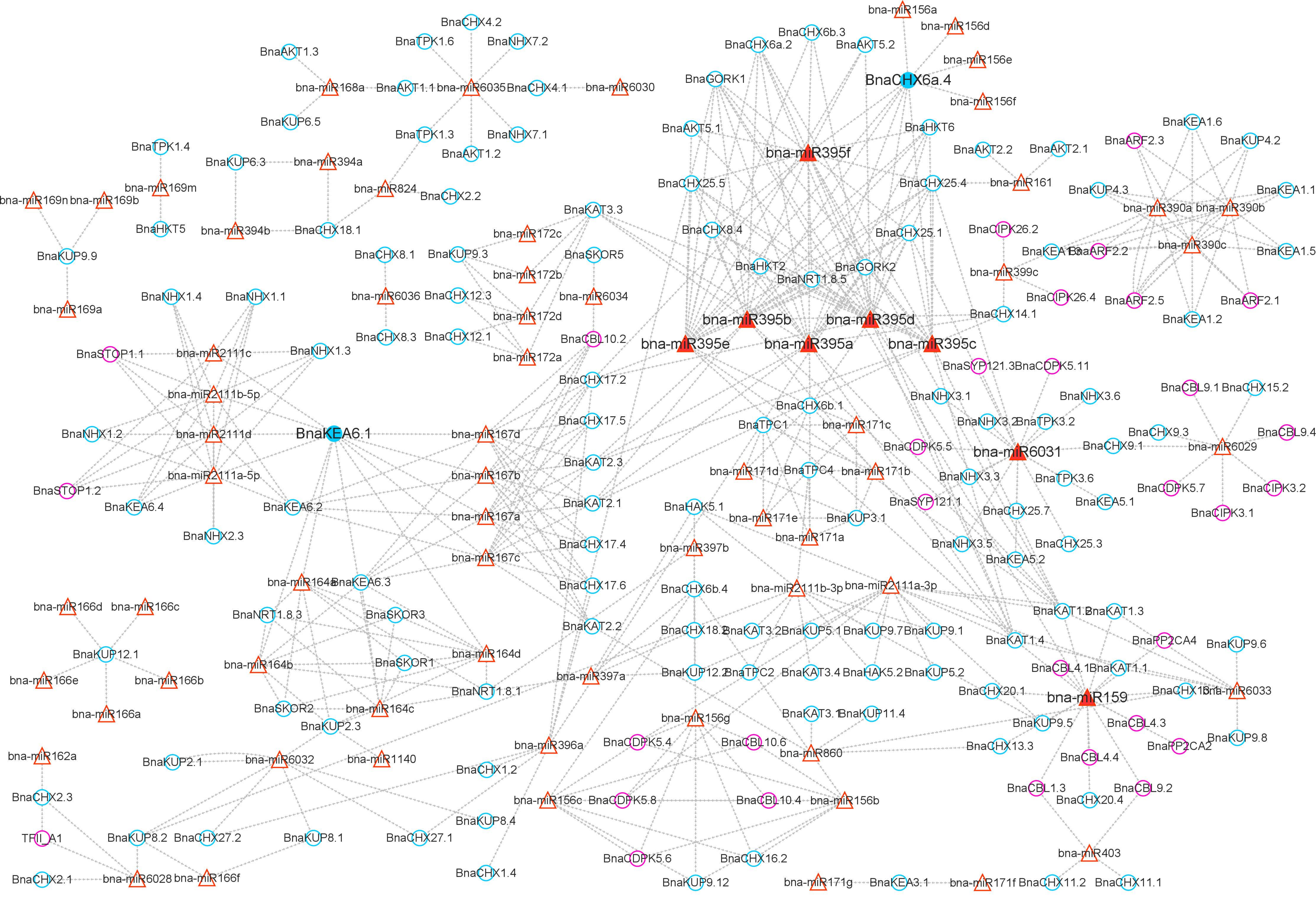

Regulation of miRNA on K utilization related genes in *B. napus*.Red triangles represent miRNAs, circles represent K utilization related genes, purple represents functional genes, blue represents regulation genes.
