# Supplementary figures and images for: The Potassium Utilization Gene Network in Brassica napus and Functional Validation of BnaZSHAK5.2 Gene in Response to Potassium Deficiency
Source: Int J Mol Sci. 2025 Jan 18;26(2):794. doi: 10.3390/ijms26020794 (PMC11765689; doi:10.3390/ijms26020794)

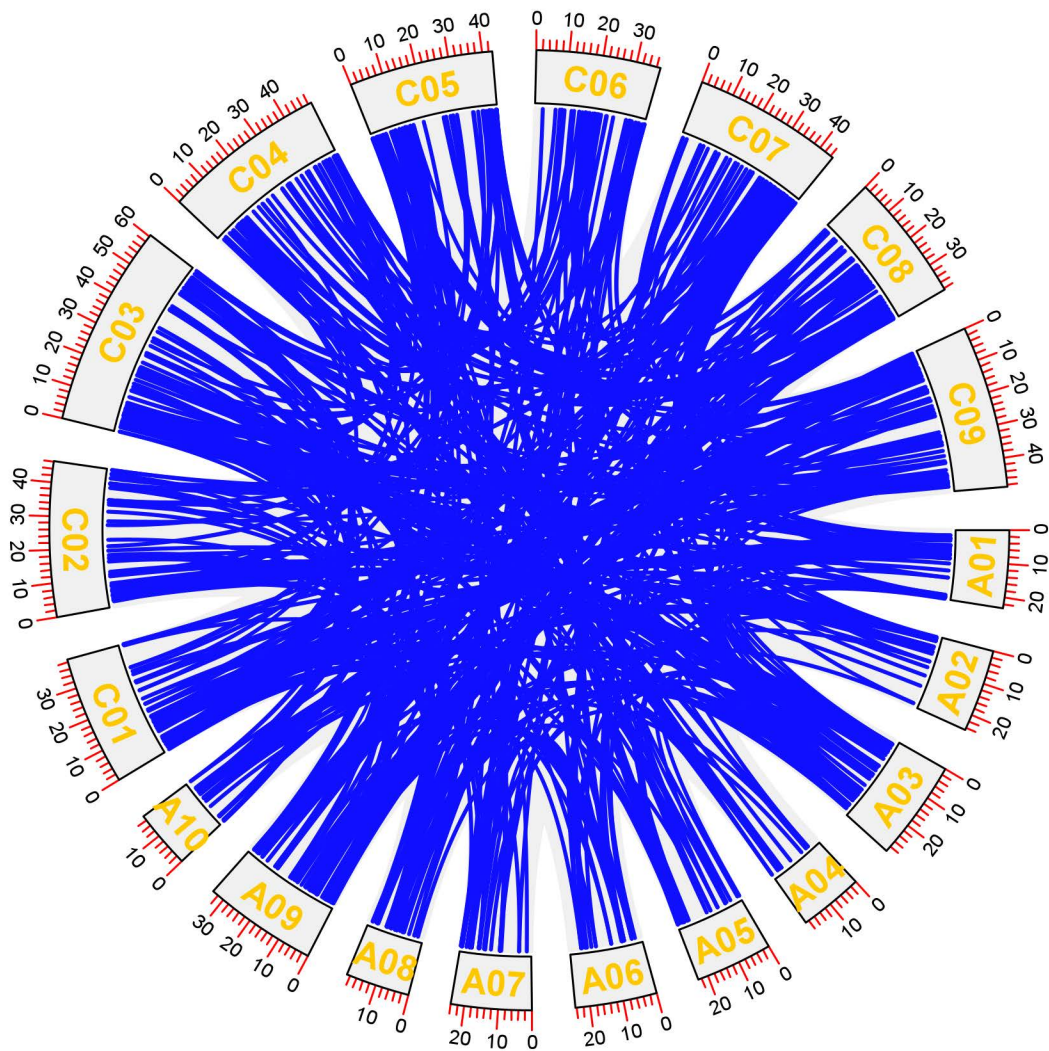

Intraspecific collinearity analysis of *B.napus*

Supplement: Supplementary file 1 [file ijms-26-00794-s001.zip › Supplementary Figure S2.pdf]

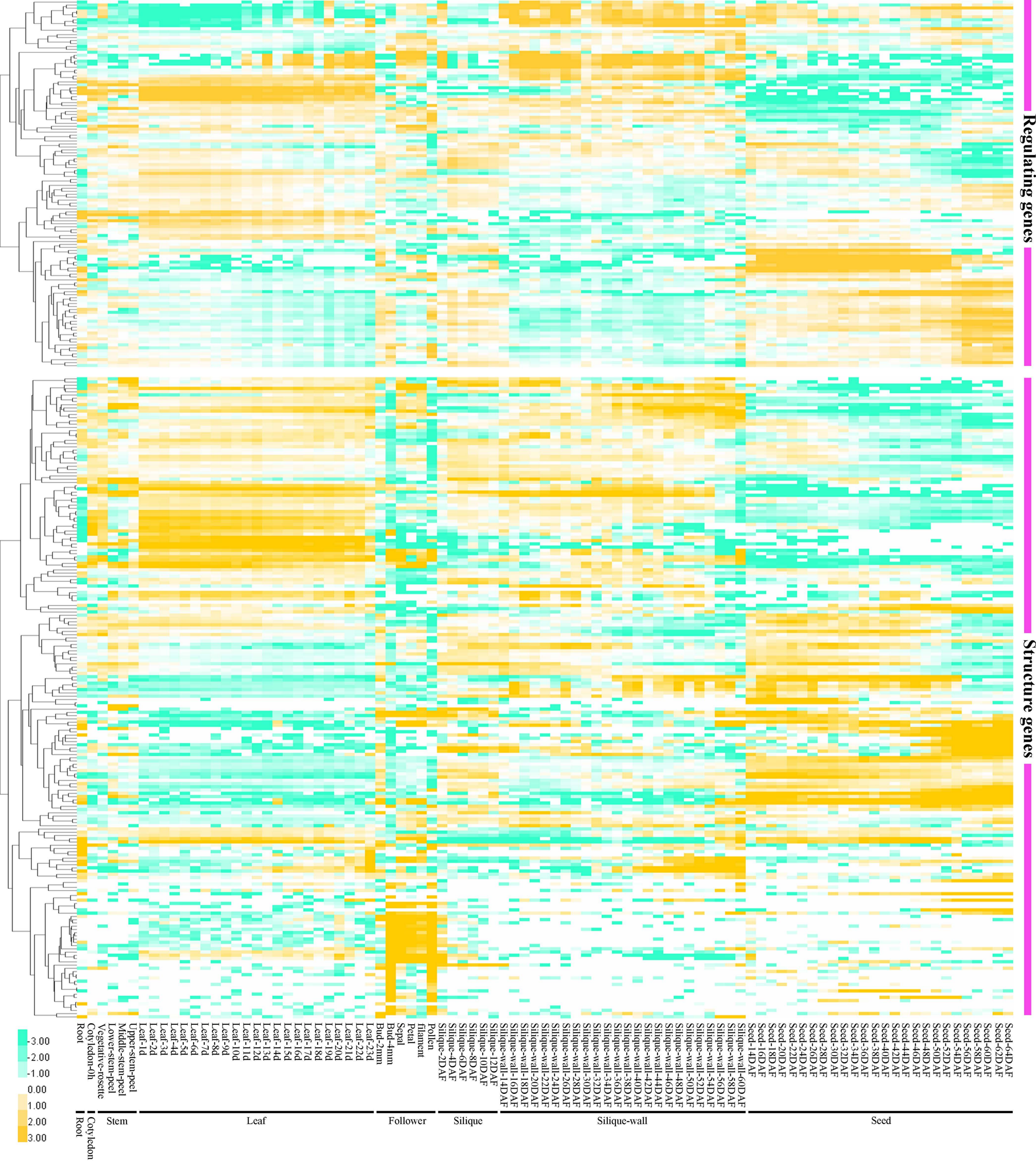

Tissue expression profile of K utilization related genes in *B. napus*

Supplement: Supplementary file 1 [file ijms-26-00794-s001.zip › Supplementary Figure S4.pdf]
